# Rectus abdominis muscle transplant for repair of abdominal wall defects required for cancer resections: Case report

**DOI:** 10.1016/j.ijscr.2019.08.014

**Published:** 2019-08-19

**Authors:** Paul H. Sugarbaker

**Affiliations:** Program in Peritoneal Surface Malignancies, MedStar Washington Hospital Center, 106 Irving St., NW, Suite 3900, Washington, DC, 20010, USA

**Keywords:** Appendiceal cancer, Ovarian cancer, Laparoscopy port site, Muscle transplant, Tumor cell entrapment, Hernia mesh

## Abstract

**Background:**

Cancer cells from peritoneal metastases may gain access to tissues of the abdominal wall. If laparotomy or laparoscopy is performed in a patient with peritoneal metastases, the abdominal incision or trochar sites may become seeded by cancer cells. Progression of disease may require resection if cancer at other sites can be controlled.

**Methods:**

Metastases in an appendectomy incision occurred from peritoneal metastases of an appendiceal mucinous adenocarcinoma. The clinical features and treatment strategy of this condition in a single patient was studied.

**Results:**

In this patient, cytoreductive surgery (CRS) and hyperthermic intraperitoneal chemotherapy (HIPEC) was used to eradicate the peritoneal metastases. The cancer isolated within the appendectomy site was resected leaving a large abdominal wall defect. The defect was closed by a rectus abdominis muscle flap dissected from the contralateral side of the abdominal wall. No mesh was required.

**Conclusions:**

Abdominal wall defects from resection of cancerous masses of the abdominal wall can be closed with a contralateral rectus abdominis muscle transplant in the absence of mesh.

## Highlights

• Cancer progression within a laparotomy incision or laparoscopy port site may create a large abdominal wall defect.
• In patients with abdominal or pelvic cancer, mesh repair of a hernia may be contraindicated.
• Autologous tissue to repair a large abdominal wall hernia may provide a superior result.
• A rectus abdominus muscle transplant can be used to repair a large contralateral abdominal wall hernia defect.

## 1 Introduction

When cancer progresses within the tissues of the abdominal wall, it must be resected. This involvement usually involves all layers of the abdominal wall because it is most commonly associated with surgical access to the peritoneal cavity which must transect tissues from skin to peritoneum. The cancer progression within the layers of the abdominal wall results from spread of disease from peritoneal metastases. By tumor cell entrapment cancer cells from the abdomen or pelvis are distributed along the path of surgical trauma, then implant with high efficiency and progress [1,2].

The two most common surgical procedures that cause abdominal wall cancerous masses are laparotomy or laparoscopy. Also, transabdominal needle biopsy of a cancerous lesion can seed the abdominal wall by cancer but this is less common. If there are free cancer cells within the peritoneal cavity these cells can seed the tissues of the abdominal all and progress as a cancerous mass within an abdominal incision or within a laparoscopy port site [3,4].

These recurrences routinely involve all layers of the abdominal wall except the skin. They are not well contained in that the entire length of the abdominal incision may be seeded and goes on to develop an abdominal wall recurrence. Also, all laparoscopy sites except those within the linea alba go through the many muscle and fascial layers of the abdominal wall. The trochar does not move straight through these multiple layers but may take a circuitous path through the abdominal wall resulting in a poorly defined mass. Also, cancer progression along muscle fascicles occurs causing extension of the process within the muscle itself [5].

These tumor masses within the abdominal wall may not be clinically relevant if the patient has the progression of peritoneal metastases and multiple other sites of metastatic disease. However, if control of intraabdominal cancer can be achieved with cytoreductive surgery (CRS) and perioperative chemotherapy including hyperthermic intraperitoneal chemotherapy (HIPEC) these masses must be definitively resected [6].

Cancerous masses within the abdominal wall are usually large to moderate in size before they are referred for resection. The defect is such that component separation of the abdominal wall musculature will not facilitate reconstruction following resection. An interposition mesh can be used to fill the defect. In this patient autologous tissues was successfully used without foreign material (hernia mesh) to close the large abdominal wall defect. A rectus abdominis muscle from the contralateral side of the abdomen was sutured into the defect with satisfactory short-term and long-term results.

Data on this patient was prospectively recorded and then retrospectively reviewed at an academic institution. This research work has been reported in line with the SCARE criteria [7]. This study was registered as a case report on the www.researchregistry.com website with UIN 4799.

## 2 Patient presentation

September 1991, after a diagnosis of appendicitis this 36 year old woman had an appendectomy and then a right colectomy. Right colon resection was through a transverse incision. Pathology showed well-differentiated adenocarcinoma of the appendix [8].

January 1992, abdominal re-exploration was performed for tumor progression. A greater omentectomy was performed. Pathology showed well-differentiated mucinous neoplasm compatible with a primary appendiceal tumor. Exploration was through a midline incision and the prior right transverse incision was left intact.

April 1992, gross progression of peritoneal metastases from mucinous adenocarcinoma was observed by abdominal CT. The midline abdominal incision was resected and widely reopened from xiphoid to pubis. The right transverse incision was left in place and thought to not be involved. A visceral sparing cytoreductive surgery was performed with a peritoneal cancer index of 11 [9]. The completeness of cytoreduction score was 1 [9]. The patient had early postoperative intraperitoneal chemotherapy with mitomycin C and 5-fluorouracil. Postoperatively, the patient received 4 cycles of combined intravenous mitomycin C and intraperitoneal 5-fluorouracil [10].

Soon after these treatments were completed the patient noted pain from an enlarging mass progressing within the scar of the old right-sided transverse abdominal incision. CT showed the mass increasing in size. The mass was painful requiring narcotic analgesia.

January 1993, through an extension of the right transverse incision an en bloc resection of the skin, subcutaneous tissue, abdominal musculature, preperitoneal fat and peritoneum was performed. The resection required a circular abdominal wall defect that measured 12 cm in diameter. Skin only was closed and no effort to close the abdominal wall defect with mesh was made. The patient was instructed to wear an external abdominal binder which was well tolerated.

**Fig. 1:**
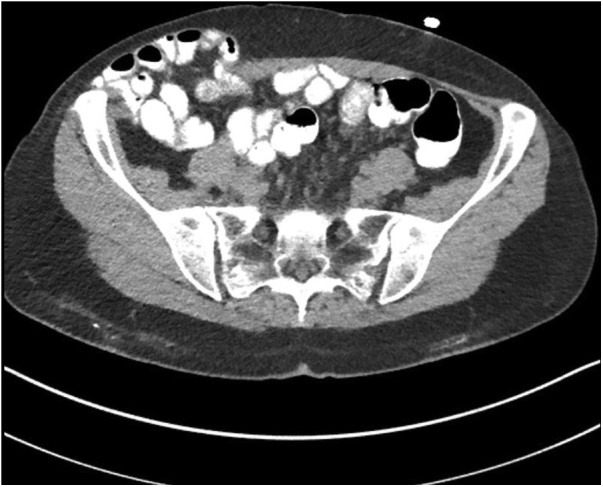
CT cut through the hernia in the right lower quadrant. By CT, it measured 8 cm in diameter. The skin and some subcutaneous tissue were intact over the hernia defect.

In 2016, the patient suffered two episodes of small bowel obstruction. On 11/28/2016 a CT of the abdomen was obtained (Fig. 1). A large hernia defect filled by bowel was obvious. No evidence of appendiceal mucinous neoplasm in the abdomen, pelvis, or abdominal wall was seen on this CT.

**Fig. 2:**
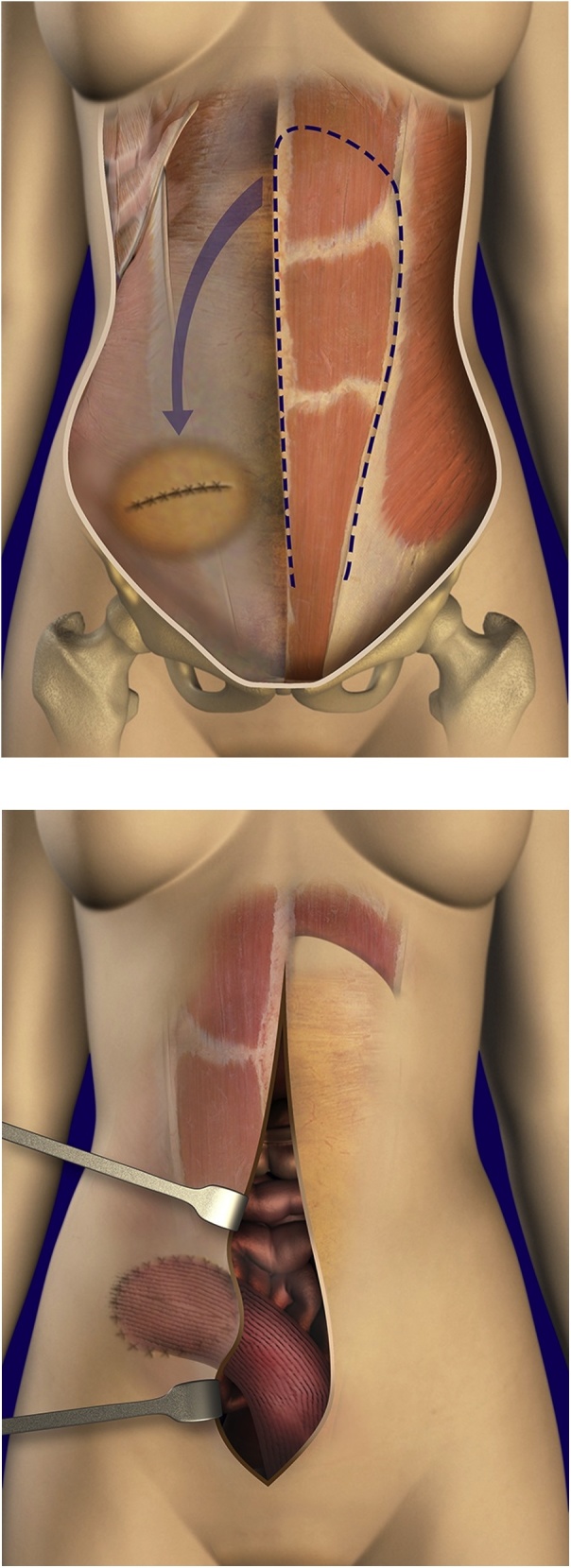
Rectus abdominis muscle transplant. Top: The rectus muscle and posterior rectus sheath beneath are mobilized. Bottom: After dividing the muscle superiorly, it is rotated to fill the contralateral hernia defect and circumferentially sutured in place.

December 2016, the long midline abdominal incision was opened. The peritoneal cavity was explored and found to be clear of mucinous tumor. The intestines were removed from the hernia defect and all adhesions lysed. No tumor was present. The hernia defect was measured 18 cm in length and 12 cm in width. To close the defect the entire left rectus muscle was resected from beneath the left anterior rectus sheath taking care to leave the posterior rectus sheath intact on the rectus muscle. The rectus muscle and posterior rectus sheath were separated from the costal margin and the superior deep epigastric vessels ligated. The inferior deep epigastric vessels were maintained intact (Fig. 2, top).

The left rectus abdominis muscle transplant was moved from left to right into the hernia defect. Working from within the abdomen individual 0 Prolene sutures (Ethicon, Indianapolis, IN) were placed through the full thickness of the abdominal muscle and fascia at the 4 quadrants of the edge of the hernia defect and then through the transplanted rectus muscle and posterior rectus sheath. Space between sutures was bisected with another 0 Prolene suture until sutures were 1–2 cm apart and evenly spaced. The peritoneum from the edges of the hernia defect was used to cover the knots of the nonabsorbable sutures (Fig. 2, bottom). The midline abdominal incision was closed with a running 0 Prolene suture in a routine manner. The patient recovered without incident.

January 2019, repeat CT scans and physical examination show no defect at the hernia repair site on the right side of the abdomen and no bulge of the left side of the abdominal wall from which the left rectus muscle and posterior rectus sheath had been removed. No further episodes of bowel obstruction have occurred.

## 3 Discussion

Often a large circular hernia defect is closed using an interposition graft of strong synthetic mesh. I elected not to use a mesh at the time of resection of the mass in January 1993 or with repair of the hernia in December 2016. At both of these explorations no tumor was seen. However, recurrence of mucinous adenocarcinoma continues to be a potential problem after the initial cancer resection from the abdominal wall. In patients with hernia who also carry a diagnosis of abdominal or pelvic malignancy, hernia mesh may be considered contraindicated [11]. Cancer does have a propensity to infiltrate the interstices of the mesh. Also, the mesh creates an inflammatory response with the accumulation of extensive fibrous tissue. Placement of mesh makes reoperative surgery difficult and dangerous should the cancer recur and require further interventions.

The rectus abdominis muscle transplant when used to repair a large contralateral hernia defect is not only muscle but also the posterior rectus sheath. These two structures are maintained as a unit to fill the hernia defect. The posterior rectus sheath adds strength to the muscle that holds the strong nonabsorbable sutures. The transplanted tissue if it does not develop defects should become stronger over time because it is autologous tissue.

A potential criticism of the rectus abdominis muscle transplant is a weaker abdominal wall because the rectus muscle is transplanted to the contralateral side of the abdomen. In the multiple rectus abdominis muscle transplants the weakness of the abdominal wall and anterior rectus sheath has not been a problem. The transplanted rectus abdominis muscle has been used for filling the abdominal or pelvic spaces that often remain after major cancer resections. If the entire kidney and psoas muscle must be resected, the ipsilateral rectus muscle can be used to fill this space. If there is an extensive resection within the pelvis, one or both rectus muscles can be used to resurface the pelvic floor [12]. To this point in time the anterior rectus sheath has always stayed intact and no abdominal wall defects have occurred.

In patient 1, a staged procedure was performed with resection of the mass within the abdominal incision and hernia repair at a later time. This delay indicated that the malignancy was not likely to recur. Also, the muscular/fascial edges of the hernia defect had become fibrotic and held the circumferential individual sutures well.

## Sources of funding

Data management and secretarial support provided by Foundation for Applied Research in Gastrointestinal Oncology.

## Ethical approval

Local IRB-approval for this case report was not required:

MedStar Health Institutional Review Board has determined that a case report of less than three (3) patients does not meet the DHHS definition of research (45 CFR 46.102(d)(pre-2018)/45 CFR 46.102(l)(1/19/2017)) or the FDA definition of clinical investigation (21 CFR 46.102(c)) and therefore are not subject to IRB review requirements and do not require IRB approval.

This case report is of 1 patient.

## Consent

Written and signed consent was obtained from the patient.

## Author’s contribution

Paul H. Sugarbaker, MD: study concept or design, data collection, data analysis or interpretation, writing the paper.

## Registration of research studies

This study was registered as a case report on the www.researchregistry.com website with UIN 4799.

## Guarantor

Paul H. Sugarbaker, MD.

## Provenance and peer review

Not commissioned, externally peer-reviewed.

## Declaration of Competing Interest

Paul H. Sugarbaker has no conflicts of interest to declare.
